# Supplementary material for: Segment anything small for ultrasound: Enhancing segmentation with non-generative augmentation
Source: PLOS Digit Health. 2026 Apr 8;5(4):e0001309. doi: 10.1371/journal.pdig.0001309 (PMC13061201; doi:10.1371/journal.pdig.0001309)
Supplement: S1 Fig — (PDF) [file pdig.0001309.s001.pdf]

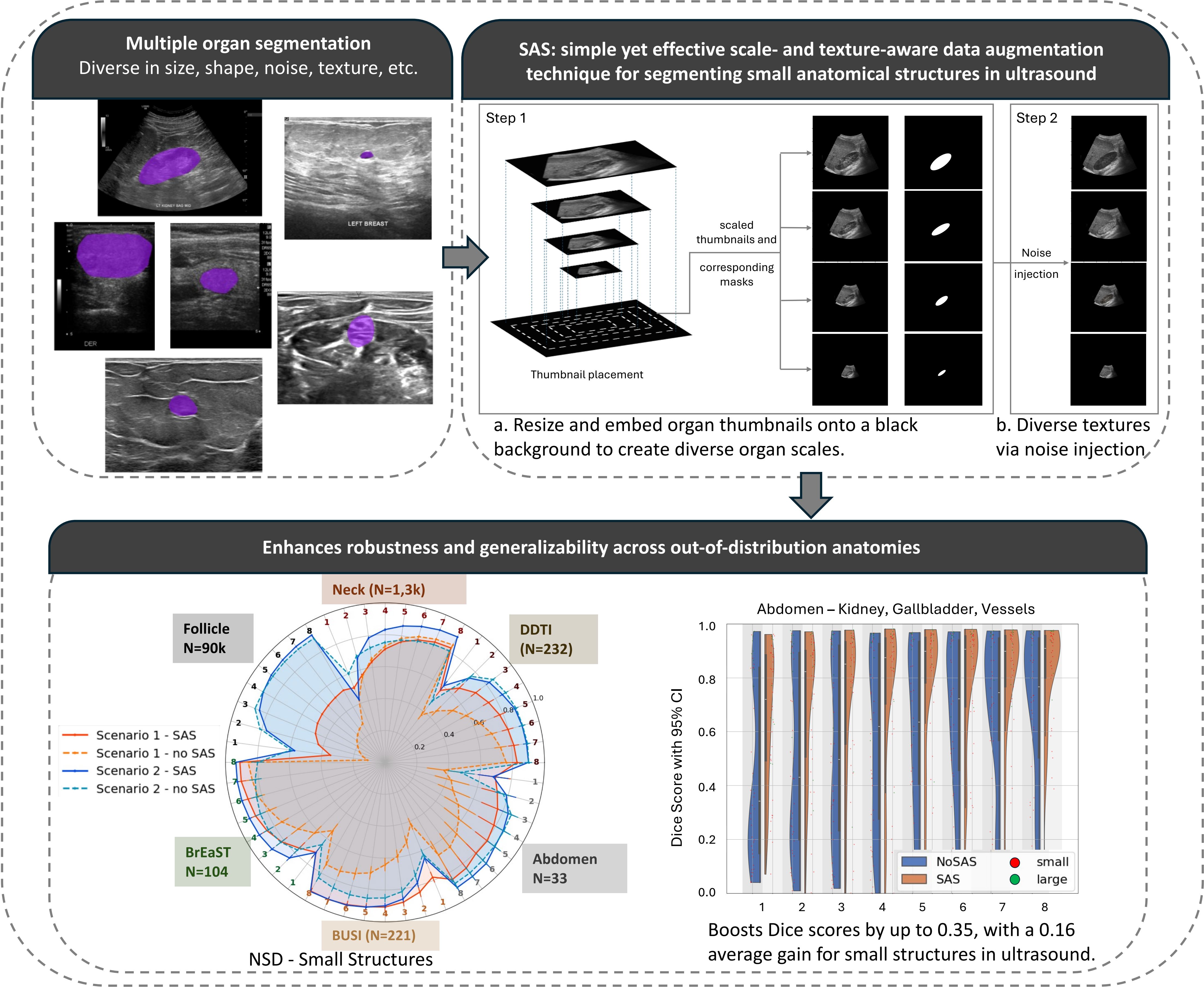

Multiple organ segmentation
Diverse in size, shape, noise, texture, etc.
LEFT BREAST
SAS: simple yet effective scale- and texture-aware data augmentation technique for segmenting small anatomical structures in ultrasound
Step 1
Thumbnail placement
scaled thumbnails and corresponding masks
Step 2
Noise injection
a. Resize and embed organ thumbnails onto a black background to create diverse organ scales.
b. Diverse textures via noise injection
Enhances robustness and generalizability across out-of-distribution anatomies
Neck (N=1,3k)
Follicle N=90k
DDTI (N=232)
Scenario 1 - SAS
Scenario 1 - no SAS
Scenario 2 - SAS
Scenario 2 - no SAS
BrEaST N=104
Abdomen N=33
BUSI (N=221)
NSD - Small Structures
Abdomen – Kidney, Gallbladder, Vessels
Dice Score with 95% CI
NoSAS
SAS
small
large
Boosts Dice scores by up to 0.35, with a 0.16 average gain for small structures in ultrasound.
