# Supplementary material for: Segment anything small for ultrasound: Enhancing segmentation with non-generative augmentation
Source: PLOS Digit Health. 2026 Apr 8;5(4):e0001309. doi: 10.1371/journal.pdig.0001309 (PMC13061201; doi:10.1371/journal.pdig.0001309)
Supplement: Attachment — Submitted filename: PLOS_ResponseToReviewers.pdf [file pdig.0001309.s002.pdf]

Dear Editor,

We thank you and the reviewers for their valuable feedback. In this revision, we have implemented several key updates:
(1) we improved the clarity and consistency of Figures 1 and 3 (old 2), including revised visualizations and corrected noise examples; (2) we added a new ablation study that explicitly evaluates the individual and combined contributions of scale and noise perturbations in the proposed SAS strategy; (3) we strengthened methodological justifications and empirical analyses, including object-size thresholding and background modeling; and (4) we revised the language throughout the manuscript to improve clarity, tone, and reproducibility.

In the following, we provide detailed point-by-point responses to the concerns and suggestions from the reviewers.

Reviewer #1: This article investigates and proposes a method called SAS for data augmentation, with the research demonstrating that the SAS-based approach effectively enhances the performance of image segmentation, particularly showing greater effectiveness for smaller targets.

The overall structure of the article is clear and well-supported by evidence. However, there are a few specific areas where additional clarification or improvement could aid reader comprehension:

Q1. In Figure 3, Scenarios 1 and 2 use colors that are too similar to represent SAS and no-SAS samples, making comparison challenging. Since Section 3.2 specifically discusses how no-SAS samples cluster tightly in Scenario 1, using more distinct colors would better highlight the separation between SAS and no-SAS distributions as observed through t-SNE visualization.
A1: Thank you for this valuable suggestion. We have revised Figure 3 (now Figure 4 after the insertion of a new Figure 2) to improve visual distinction between SAS and no-SAS samples. In the updated figure, no-SAS samples are now shown in red for Scenario 1 and green for Scenario 2, while SAS samples are displayed using clearly contrasting colors.

Q2. The SAS method achieves data augmentation through two steps: image scaling and noise addition. While both steps contribute positively—scaling improves small target detection and noise enhances robustness—the combined effect shown in Figure 3 via t-SNE doesn't clarify whether the dimensionality reduction reflects the impact of scaling, noise addition, or both. A visualization comparing feature distributions after each step separately would better illustrate their individual contributions.
A2: We thank the reviewer for raising this important point. We agree that disentangling the effects of scale and noise perturbations is necessary. Rather than relying on t-SNE, which is a qualitative visualization and sensitive to hyperparameter choices, we address this directly by adding Section 3.5 (Ablation Study). This section presents a controlled ablation comparing scaling-only, noise-only, and the full SAS pipeline, evaluated using quantitative performance metrics and feature-

space diversity analyses. The results, reported in Section 3.5 and Figure 10, quantitatively demonstrate the distinct and complementary contributions of scale and noise perturbations.

Q3. The four types of noise (Speckle, Gaussian, Salt and Pepper, Poisson) used in Step 2 do correspond to typical ultrasound noises. However, in Figure 1, the image added at the bottom of Step 2 appears to be a mosaic image rather than one of the specified noise types. This inconsistency should be reviewed by the authors.
A3: Thank you for pointing out this inconsistency. In the original version of Figure 1, the texture shown in Step 2 was intended for illustrative purposes only and did not correspond to one of the explicitly listed noise models, which could lead to confusion. We have revised the figure to replace this illustrative texture with images generated using the actual noise perturbations described in the method (Speckle, Gaussian, Salt-and-Pepper, and Poisson), and we now explicitly label the noise type applied in the figure.

The article makes significant contributions but these specific refinements would strengthen its communication of findings.

Reviewer #2:
Q1. phrases such as "emerges as a powerful tool" read as promotional; please revise to reflect performance association rather than definitive superiority.
A1: We thank the reviewer for this suggestion and have revised the text accordingly to avoid promotional phrasing and to emphasize performance association.

Q2. Justify the 3% threshold for "small structures" (Methods, Section 2.1.3, lines 149–154): Please provide a citation or empirical rationale for this cutoff, or include a sensitivity analysis showing robustness to alternative thresholds.
A2: Thank you for pointing this out. We have added an empirical justification for the 3% threshold used to define small structures to Section 2.2.3. As shown by the distribution of relative structure areas, the data exhibit a strong concentration below 3% and a clear transition to a long-tailed regime beyond this value, making 3% a natural cutoff separating small from larger anatomies.

Q3. Expand justification for black background placement (Methods, Section 2.1.1, lines 131–134): Please explain why a zero-intensity background best reflects ultrasound acquisition physics and whether this risks domain shift.
A3: We thank the reviewer for raising this point on the zero-intensity background. We clarified that the black background is used to mimic ultrasound display zoom behavior—where the rendered image occupies only part of the screen and remaining pixels are zero-valued—rather than the acoustic field-of-view, and that these padded regions contain no anatomical signal, contribute minimal gradients during backpropagation, and do not introduce domain shift.

Q4. The manuscript would benefit from explicitly stating whether scale and noise components were tested independently, and where those results are reported

A4: We thank the reviewer for this suggestion. As detailed in our response to R1, we have now explicitly evaluated scale and noise perturbations independently and jointly through a dedicated ablation study. The results are reported in Section 3.5 (Ablation Study) and summarized in Figure 10, which compares No SAS, Noise only, Scale only, and SAS (Scale + Noise) across clicks and object sizes. We have updated the manuscript to clearly describe this experimental setup.

Q5. The explanation for underperformance on large breast tumors is reasonable, but please add a quantitative comparison to show the magnitude and consistency of degradation
A5: Thank you for the helpful suggestion. We have added a quantitative comparison to characterize the magnitude and consistency of underperformance on large breast tumors in the Section Discussion and Conclusion, with the corresponding quantitative results shown in Figure 5.

Q6. The nerve dataset shows minimal improvement; please add a brief explanation of why SAS may be less effective for elongated, low-contrast structures.
A6: We thank the reviewer for this insightful comment. We have added a brief explanation noting that SAS is less effective for elongated, low-contrast structures such as nerves in Section 3.3; because SAS primarily augments scale without explicitly modeling deformation or long-range anatomical context, its impact on nerve segmentation is consequently more limited.

Q7. Tighten speculative language in Future Work (Discussion, lines 415–423): Please distinguish clearly between planned experiments and hypotheses, avoiding language that implies expected success.
A7: Thank you for this valuable suggestion. We have revised the Future Work section to distinguish planned evaluations from hypotheses clearly.
